# Innate Lymphocytes in Inflammatory Arthritis

**DOI:** 10.3389/fimmu.2020.565275

**Published:** 2020-09-25

**Authors:** Xunyao Wu

**Affiliations:** The Ministry of Education Key Laboratory, Department of Rheumatology and Clinical Immunology, Peking Union Medical College Hospital, Clinical Immunology Center, Chinese Academy of Medical Sciences and Peking Union Medical College, Beijing, China

**Keywords:** Inflammatory arthritis, innate-like lymphocytes, innate lymphoid cells, inflammatory cytokine, NK cells

## Abstract

Inflammatory arthritis (IA) refers to a group of chronic diseases, including rheumatoid arthritis (RA), psoriatic arthritis (PsA), ankylosing spondylitis (AS), and other spondyloarthritis (SpA). IA is characterized by autoimmune-mediated joint inflammation and is associated with inflammatory cytokine networks. Innate lymphocytes, including innate-like lymphocytes (ILLs) expressing T or B cell receptors and innate lymphoid cells (ILCs), play important roles in the initiation of host immune responses against self-antigens and rapidly produce large amounts of cytokines upon stimulation. TNF (Tumor Necrosis Factor)-α, IFN (Interferon)-γ, Th2-related cytokines (IL-4, IL-9, IL-10, and IL-13), IL-17A, IL-22, and GM-CSF are involved in IA and are secreted by ILLs and ILCs. In this review, we focus on the current knowledge of ILL and ILC phenotypes, cytokine production and functions in IA. A better understanding of the roles of ILLs and ILCs in IA initiation and development will ultimately provide insights into developing effective strategies for the clinical treatment of IA patients.

## Introduction

Inflammatory arthritis (IA) describes a group of autoimmune-associated diseases with sustained chronic inflammation that eventually result in disability and decreased quality of life. IA includes rheumatoid arthritis (RA), psoriatic arthritis (PsA), ankylosing spondylitis (AS), and other spondyloarthritis (SpA), which have similar and different clinical features (1). RA is characterized by joint inflammation, osteoclast-mediated cartilage, local bone destruction and usually affects the limbs first. Autoantibodies against citrullinated peptide (ACPA) and rheumatoid factor (RF) can be detected in the serum and inflamed synovial membrane of nearly two-thirds of RA patients (2). PsA is a seronegative, systematic inflammatory joint disease, and up to 30% of PsA patients have psoriasis. PsA displays heterogeneous musculoskeletal characteristics, including peripheral arthritis, entheses, dactylitis, and axial skeleton and dermal manifestations (3). AS is considered a genetic immune-mediated arthritis that has a strong correlation with *HLA-B27*. In As patients, the joints of the spine are the most affected (4). AS also displays a number of other clinical characteristics, including peripheral arthritis, ligament, enthesis attachment and inflammatory bowel disease (IBD). Other SpA includes a subgroup of juvenile idiopathic arthritis, reactive arthritis and IBD-associated arthritis (5). It is widely accepted that IA is strongly associated with immune disorders, but the current understanding of the immune pathogenesis of IA is still limited. Uncovering how inflammation is resolved could help the development of innovative strategies for clinical IA therapy.

Innate lymphocytes, including innate-like lymphocytes (ILLs) and innate lymphoid cells (ILCs), play important roles in the initiation of host immune responses against intracellular and extracellular pathogens or self-antigens. ILLs such as natural killer T (NKT) cells, mucosal-associated invariant T (MAIT) cells, γδ T cells, B-1 cells and marginal zone B (MZB) cells preferentially utilize specific TCR or BCR genes and respond immediately upon antigen exposure (6). ILCs are particularly abundant at barrier surfaces. These cells lack T cell or B cell receptors and do not undergo clonal selection. The ILC family consists of three major groups: Group 1 ILCs include conventional natural killer (NK) and Interferon (IFN)-γ-secreting ILC1s; Group 2 ILCs (ILC2s) mainly produce interleukin (IL)-4, IL-5, IL-9, and IL-13; and Group 3 ILCs (ILC3s), including lymphoid tissue inducer (LTi) cells, predominantly secrete IL-17 and IL-22 (7). In addition to their roles in orchestrating inflammatory responses to pathogens, innate lymphocytes also directly contribute to inflammation resolution and tissue homeostasis maintenance (8–10). In this review, we discuss the role of innate lymphocytes in IA and how current or potential new clinical approaches could be applied to modulate persistent inflammatory responses in IA patients.

## Innate Lymphocytes-Associated Cytokines in IA

Cytokines have been demonstrated to play critical roles in IA, and anti-inflammatory cytokine therapies are therefore attractive therapeutic strategies. One of the key features of innate lymphocytes is their ability to produce large amounts of cytokines rapidly upon stimulation. Cytokine production mediates subsequent adaptive immune cell activation and raises the possibility of continuous cytokine production in self-reinforcing stimulatory loops during chronic inflammation (11). Moreover, proinflammatory cytokines can amplify local inflammation and further promote the generation of matrix-degrading proteolytic enzymes or reactive oxygen species, which result in organ damage and clinical symptoms of autoimmune diseases (12). Therefore, proper understanding of innate lymphocyte-associated cytokines in IA would help us gain a better understanding of innate lymphocytes in IA. The roles of innate lymphocytes-associated cytokines are summarized in Table 1.

**TABLE 1:** A Summary of innate lymphocytes-associated cytokines in inflammatory arthritis.

Cytokines	Role	Mechanisms	References
TNF-α	Pathogenic	Induce prolonged IL-6 production and NF-κB activation in FLSs; Inhibit osteoblast differentiation and associated with bone destruction.	(14, 15)
IFN-γ	Dual roles	(1) Protective: reduce cell death; inhibiting IL-1β-induced MMP synthesis by RA FLS; inhibit Th17 cell development and function. (2) Pathogenic: activate IL-17 induced pathology or directly effects skin and bone cells in PsA; drive tissue remodeling in RA.	(16–22)
IL-4/IL-13	Protective	Prevent osteoclast formation; IL-4 also suppresses TNF-α-mediated osteoclastogenesis	(23–26)
IL-9	Dual roles	(1) Pathogenic: promote pathological T cell proliferation; prolong the survival of neutrophils and increase their MMP9 expression; promote Th17 cell differentiation; drive γδ T cell expansion and activation. (2) Protective: ILC2-derived IL-9 mediates chronic inflammation resolution and protects against bone loss.	(27–30)
IL-10	Protective	Exhibit a suppressive effect on Th17 cell activation; Induce the generation of Foxp^3+^ Tregs in RA.	(31–34)
IL-17	Pathogenic	Promote angiogenesis; Induce monocyte migration; Induce inflammatory cytokines, chemokines and MMP secretion in FLS; Promotes neutrophil migration.	(36–42)
IL-22	Pathogenic	Enhance FLS expansion and MMP production; Exhibit osteoclastogenic effects; Stimulate IL-1β production and promotes neutrophils infiltration in joints; Maintain GC and promote autoantibody secretion.	(43–47)
GM-CSF	Pathogenic	Support monocytes differentiate into CD1c^+^ DCs and involve in FLS proliferation; Activates and triggers proinflammatory responses in CCR2^+^Ly6C^hi^ monocytes.	(50–53)

## Th1-Related Cytokines: TNF-α and IFN-γ

It is widely accepted that uncontrolled TNF production is associated with IA development. The TNF inhibitors infliximab, etanercept, and adalimumab are current standard clinical treatments for IA (13). TNF-α is a key driver of sustained synovial inflammation by inducing prolonged IL-6 production and NF-κB activation in FLSs (14). Moreover, TNF-α is also a well-known inhibitor of osteoblast differentiation and is associated with bone destruction in IA (15).

Interferon-γ is a classic Th1-related proinflammatory cytokine and has been identified as the most important agent for the regulation of inflammation (16). IL-12 enhances the production of IFN-γ or other important IFN-γ inducers, such as IL-23, IL-18, and IL-27 (17). IFN-γ plays dual roles in IA. IFN-γ exhibits a protective effect in IA by the following mechanisms: (1) reducing inflammatory cell death by targeting necroptosis (18); (2) inhibiting IL-1β-induced matrix metalloproteinase (MMP) synthesis by RA FLSs, thereby limiting cartilage degradation (19); and (3) and inhibiting Th17 cell development and suppressing Th17 cell effector functions (20). On the other hand, in PsA, IFN-γ promotes the development of PsA either by activating antigen-presenting cells (APC) to further contribute to IL-17 induced pathology or directly effects skin and bone cells (21). Moreover, in another study performed by Karonitsch et al., they revealed unique effects of IFN-γ in driving tissue remodeling in arthritis (22).

## Th2-Related Cytokines: IL-4, IL-9, IL-10 and IL-13

IL-4, IL-9, IL-10, and IL-13 are Th2-related cytokines that are associated with anti-inflammatory and antiosteoclastogenesis effector functions in IA. IL-4 and IL-13 share common IL-4Rα and STAT6 signaling pathways (23). IL-4/IL-13 secretion and STAT6 signaling activation play crucial roles in inhibiting IA development (24). IL-4 and IL-13 are also known to induce osteoblasts to produce osteoprotegerin (OPG), an inhibitor that prevents osteoclast formation. IL-4 induces a stronger effect on OPG production than IL-13 (25). IL-4 also suppresses TNF-α-mediated osteoclastogenesis by inhibiting stomal cell RANKL expression and directly affects stromal cells and osteoclast precursors (26).

IL-9 concentrations are higher in the synovial fluid (SF) of RA and PsA patients than in that of osteoarthritis (OA) patients. IL-9 could promote pathological T cell proliferation through the PI3K/Akt/mTOR signaling pathway in the synovium in IA (27). Synovial IL-9 could also prolong the survival of neutrophils, increase their MMP9 expression, and promote Th17 cell differentiation by inducing RORγt and STAT3 phosphorylation (28). The IL-9/IL-9R axis drives γδ T cell expansion and activation in PsA (29). However, the role of IL-9 in IA has been a subject of controversy, and recent research has demonstrated that ILC2-derived IL-9 mediates chronic inflammation resolution and protects against bone loss (30).

IL-10, a well-known immunosuppressive cytokine, can be produced by all leukocyte subsets and restrains IA development (31). IL-10-deficient mice display more severe arthritis than wild-type (WT) mice, demonstrating that IL-10 is able to ameliorate IA disease severity (32). In collagen-induced arthritis (CIA), macrophages from IL-10^–/–^mice show enhanced IL-17 and RORγt expression compared with those of WT mice (33). IL-10 exhibits a suppressive effect on Th17 cell activation and induces the generation of Foxp3^+^ Tregs in RA patients (34).

## Th17-Related Cytokines: IL-17A and IL-22

IL-17A is clearly critical in IA development and plays a role in many stages of IA. It is well established that IL-23 is a major inducer of IL-17A secretion and that the IL-23-IL-17A axis plays a key role in IA (35). IL-17A promotes IA in many respects. In the pathogenesis of RA, IL-17A promotes angiogenesis and induces human lung microvascular endothelial cell (HMVEC) migration through the PI3K/AKT1 pathway (36). IL-17 induces monocyte migration into the joints by binding to IL-17RA, and this migration is mediated through p38 MAPK signaling (37). Moreover, IL-17A induces inflammatory cytokines (including IL-6, TNF, IL-1, and RANKL) (38, 39), chemokines and MMP secretion in FLS (40). Furthermore, IL-17A synergizes with cobalt chloride (CoCl_2_), a hypoxia mimetic, to exacerbate osteoclast-mediated bone erosion through the activation of the RANKL/NFκB/NFATc1 signaling pathway (41). Additionally, IL-17A promotes neutrophil migration through a CXC chemokine-dependent pathway (42).

IL-22, a Th17 cytokine, belongs to the IL-10 family. The pathogenic functions of IL-22 in the joints have been described. IL-22 enhances FLS expansion and RA FLS-derived MMP1 and S100A8/A9 production (43, 44). IL-22 has osteoclastogenic effects on RA by inducing RANKL expression in FLSs, and these effects are mediated by the p38 MAPK/NF-κB and JAK-2/STAT3 signaling pathways (45). IL-22 also stimulates IL-1β production and promotes neutrophil infiltration in joints (46). In murine CIA, IL-22 is required for germinal center (GC) maintenance and might promote the generation of autoantibody-secreting plasma cells (47).

## Other Cytokines: GM-CSF

Granulocyte-macrophage colony stimulating factor (GM-CSF) was first identified as a growth factor that induces hematopoietic progenitor cell differentiation into granulocytes and macrophages (48). GM-CSF has been shown to exacerbate IA disease and is absolutely required for pain development (45, 49). In RA, GM-CSFR blockade results in myeloid cell-derived proinflammatory mediator suppression and suppression of T cell activation (50). GM-CSF supports the differentiation of a subpopulation of monocytes into CD1c^+^ synovial inflammatory dendritic cells and is involved in FLS proliferation (51, 52). Moreover, GM-CSF activates and triggers proinflammatory responses in CCR2^+^Ly6C^hi^ monocytes that mediate autoimmune-associated tissue damage (53).

## The Role of ILLs in IA

Innate-like lymphocytes include NKT cells, MAIT cells, γδ T cells, and innate-like B cells. Unlike conventional adaptive T and B cells, ILLs preferentially utilize specific TCR or BCR genes and rapidly respond to antigen stimulation. Below, we summarize the roles of ILLs in IA (Table 2).

**TABLE 2:** Functions of ILLs in inflammatory arthritis.

Subtypes	Subsets and distribution (Human)	Functions and mechanism (Mice)	References
NKT	(1) IFN-γ^+^NKT in SF of RA; (2) RORγt^+^T-bet^low^PLZF^–^ iNKT with Th17-like response in joints of PsA and other SpA.	(1) IL-17^+^ NKT promote murine arthritis; (2) CD1d-dependent NKT protect murine arthritis by dampening Th1 cell responses; (1) Dampening combined gut and joint inflammation in SpA.	(55–64)
MAIT	(1) MAIT with IL-17 phenotype in SF of RA; (2) CD8^+^IL-17^+^IL-23R^+^ MAIT in SF of PsA; (3) CD8^+^IL-17^+^IL-23R^+^IL-7R^+^ MAIT in SF of SpA.	(1) MAIT exacerbate in murine CIA model; (2) IL-23/IL-17 axis in MAIT contribute to PsA; (3) IL-7/IL-17 axis in MAIT contribute to AS and other SpA.	(66–71)
γδ17 T	(1) CCR5^+^CXCR3^+^ IL-17-producing Vδ2 T in RA; (2) TEM Vγ9^+^Vδ2^+^ IL-17-producing T cells with HLA-DR and CD86 expression in SF of RA; (3) TEMγδ17 T cells in peripheral and synovium of PsA; (4) IL-23R^+^RORγt^+^ γδ17 T cells in active AS and other SpA.	(1) In CIA murine arthritis, IL-17 producing Vγ4^+^ γδ T promoted disease development; (2) In Il1rn^–/–^ spontaneously developed arthritis, CCR2^+^Vγ6^+^ γδ17 T cells participate in disease progression.	(29, 62, 78–84)
Innate-like B	(1) Reduced B10 cells in PBMC of RA; (2) Impaired B10 cells in PBMC and SF of PsA; (3) CD19^+^CD24^hi^CD38^hi^ B10 cells decreased in PBMC and SF of SpA	(1) CII-reactive MZB cells exhibit spontaneous IgM and significant APC capacity for murine arthritis development; (2) B10 is crucial for suppression of Th1/Th17 response and induction of T regulatory type 1 cells; (3) B10 directly inhibit Th17 cells generation via reduction of STAT3 phosphorylation and RORγt expression; (1) B10 present CD1d-lipid and induced iNKT cells to secrete IFN-γ to ameliorate arthritis.	(89–99)

## NKT Cells

Natural killer T cells, which co-express T cell and NK cell receptors, are able to rapidly secrete large amounts of cytokines, including GM-CSF, IFNγ, IL-2, IL-4, IL-10, IL-13, IL-17A, and TNF, upon stimulation. NKT cell responses are modulated by glycolipid antigens such as α-Galcer, which is presented by CD1d, a non-classical MHC class I-like molecule. TCRα rearrangement in CD1d-dependent NKT cells includes Vα14-Jα18 in mice and Vα24-Jα18 in humans (54).

Previous studies have shown that the percentage of NKT cells is decreased in PBMCs of RA patients compared with healthy controls, and IFN-γ-producing NKT cells were present in the SF of RA patients (55, 56). In previous studies, NKT cells have been demonstrated to have dual functions in different stages of murine arthritis. Anti-CD1d mAb administration in DBA1/J mice or knockout of Vα14-expressing NKT cells in B6 background mice resulted in the development of arthritis with reduced severity after CIA induction, suggesting that Vα14-expressing NKT cells were effector cells in IA (57). In another antibody-induced murine arthritis model, Hye Young Kim et al. showed that IL-4- and IFN-γ-secreting NKT cells played an indispensable role at the end-stage of joint inflammation by suppressing TGF-β1 production (58). IL-17-producing NKT cells were increased with disease progression and involved in disease promotion in DBA/1 mice (59). However, in another antigen-induced CD1d KO/B6 mouse arthritis model, the lack of CD1d-dependent NKT cells induced increased joint inflammation throughout the acute phase of arthritis accompanied by an enhanced arthritogenic Th1 response. These studies suggest a protective role of CD1d-dependent NKT cells during the priming phase of the disease (60). IL-17-secreting iNKT cells in the SpA joint displayed a RORγt^+^T-bet^low^PLZF^–^ phenotype (61, 62). A markedly increased NKT cell ratio was observed and found to predict radiographic changes in AS (63). iNKT cells play a regulatory role in dampening combined gut and arthritis inflammation in SpA (64).

## Mait Cells

Mucosal-associated invariant T cells are innate T cells harboring a conserved T cell repertoire: Vα7.2-Jα33 in humans and Vα19-Jα33 in mice. MAIT cells are distributed in peripheral blood and tissues, including the liver, intestine, lung, kidney, prostate, and ovary (65). In humans, MAIT cells are universally defined as CD161^hi^CD26^hi^ and express transcription factors, including T-bet (*TBX21*), eomesodermin (*EOMES*), Blimp-1 (*PRDM1*), PLZF (*ZBTB16*), type 17 transcription factors RORγt (*RORC*), and STAT3 (*STAT3*). MAIT cells in the peripheral blood are CCR7^–^ and exhibit an effector memory phenotype (CD62L^low^CD45RO^+^CD27^+^), which reflects their poor ability to migrate into secondary lymphoid organs (65). However, these cells express high levels of the chemokine receptors CCR2, CCR5, CCR9, and CXCR6, suggesting their ability to migrate into inflamed tissues. Upon stimulation, human peripheral blood-derived MAIT cells produce IFNγ, TNFα, IL-17A, and granzyme. The cytokine profile of MAIT cells differs among different tissues in mice, with high levels of IL-17A production in the spleen and intestine but preferential expression of GM-CSF, IL-4, and IL-13 in the thymus (65).

An enrichment in IL-17-expressing MAIT cells was observed in the SF in IA and appeared to contribute to the inflammatory status in arthritis (66–68). In RA patients, elevated TNFα and IL-1β in SF stimulated the expression of CCL20, ICAM-1, and VCAM-1 on human blood vessel endothelial cells (HUVECs) to facilitate MAIT cell migration (69). The severity of CIA was ameliorated in MAIT cell-deficient mice, and reconstituting MAIT cells induced severe joint inflammation, demonstrating that MAIT cells could exacerbate arthritis. Moreover, *in vitro* stimulation of MAIT cells with IL-1β induced MAIT cell proliferation, and IL-23 promoted MAIT cell production of IL-17A (70). The majority of MAIT cells in the SF in PsA but not RA were CD8^+^ cells. CD8^+^ MAIT cells produce IL-17A, which is central to the pathogenesis of PsA. Moreover, the MAIT cells in the SF in PsA were enriched in IL-23R and proliferated upon IL-23 stimulation (71). IL-17^+^ MAIT cells in AS expressed high levels of both IL-7R and IL-23R; however, these cells only responded to FLS-derived IL-7. Activation of MAIT cells with IL-23 had almost no effect on IL-17 production (68). Taken together, these studies suggest that MAIT cells are critical in the aberrant IL-17 signaling pathway and contribute to the pathogenesis of IA.

## γδ17 T Cells

γδ T cell subsets contribute to tissue damage in various autoimmune diseases, including psoriasis-like disease, IA, colitis, and experimental autoimmune encephalomyelitis (EAE). IL-17^+^ γδ T cell subtypes are common in IA pathogenesis (72). γδ17 T cells are an innate source of IL-17A and share most phenotypic markers with Th17 cells. These cells express IL-23R, IL-17A, IL-22, and RORγt, as well as the chemokine receptors CCR6 and CCR2. These chemokine receptors are also expressed by Th17 cells and are reported to direct γδ17 T cells trafficking to the dermis (73). CCR2 promotes γδ17 T cell migration to the arthritic synovium during autoimmunity (74). Although γδ17 T cell development in the thymus requires a TCR signal, the peripheral activity of these cells could be directly activated by non-TCR signals, such as IL-23 and IL-1β (75). In mice, TCR-γ consists of six Vγ subsets, of which Vγ4^+^ and Vγ6^+^ γδ T cells are the main IL-17 producers (76). In some contexts, Vγ1^+^ γδ T cells could also secrete IL-17A. In humans, the majority of γδ T cells in peripheral blood are Vγ9^+^Vδ2^+^ T cells with distinct Th1 signatures. However, upon binding with IL-1β, IL-6, TGF-β, and IL-23 and AHR ligand polarization, Vγ9^+^Vδ2^+^ T cells differentiate into IL-17-producing γδ T cells (77).

IL-17-producing Vγ4^+^ γδT cell numbers were significantly increased in CIA-induced murine arthritis, and the depletion of Vγ4^+^ γδT cells obviously attenuated disease occurrence and severity (78). CCR2^+^Vγ6^+^ γδ17 T cells played a pathogenic role in IL-1Ra-deficient (Il1rn^–/–^) mice, an IL-17-dependent spontaneous arthritis murine model. Notably, γδT cells but not Th17 cells were the primary source of IL-17A in joints (79). Yoshinago Ito et al. demonstrated that CCR6^+^ γδ T cells were the dominant producers of IL-17 in CIA-induced murine arthritis and that these cells were induced by IL-1β plus IL-23 independent of the T cell receptor. However, these cells can hardly be detected in the joints of RA patients (80). Other studies demonstrated the presence of γδ17 T cells in the synovium of RA patients. Mo et al. showed high levels of CCR5 and CXCR3 in IL-17-producing Vδ2^+^ cells driven by the TNF-α-induced NF-κB signaling pathway in the serum of RA patients (81). Recently, TEM Vγ9^+^Vδ2^+^ T cells stimulated by isopentenyl pyrophosphate could differentiate into CD45RA^–^CD27^–^ effector memory cells (TEM) and exhibit an APC phenotype with HLA-DR and CD86 expression. These cells can recognize and present autoantigen peptides to cause excessive autoreactive CD4^+^ T cell immune responses (82). TEM Vγ9^+^Vδ2^+^ T cells had a stronger ability to secrete IL-17 than non-TEM Vγ9^+^Vδ2^+^ T cells. Subsequent findings indicated that TEM Vγ9^+^Vδ2^+^ T cells are the predominant γδ T subpopulation in the SF of RA patients (82). Expansion and activation of TEM Vγ9^+^Vδ2^+^ T cells driven by the IL-9/IL-9R axis were observed in the peripheral blood and synovium of untreated PsA patients (29). An enrichment in circulating IL-17A^+^IL-23R^+^ γδ T cells was detected in patients with active AS and sJIA (83, 84). γδ17 T cells were enriched in PsA and AS patients, and their functions promoting disease progression were modulated by the key Th17 cell transcriptional regulator RORγt (62).

## Innate-Like B Cells

Rheumatoid arthritis is also characterized by autoantibody production. Innate-like B cells can be directly stimulated by Toll-like receptors rather than through BCR and TCR signaling. These cells quickly differentiate into antibody-secreting cells that produce T cell-independent “natural”, polyreactive antibodies, as well as IL-10. Innate-like B cell subsets consist of MZB cells, B1 cells, and IL-10-producing regulatory B cells (Bregs) (85, 86). Recently, a novel B cell subset, natural killer-like B (NKB) cells, which have a CD19^+^NK1.1^+^ phenotype, was identified. These cells are present in mouse spleens and mesenteric lymph nodes and express IgM and NKp46. NKB cells secrete large amounts of IL-12 and IL-18 to subsequently activate ILC1s and NK cells (87). However, by using an array of mouse genetic models, Eric Vivier et al. demonstrated that NKB cells were not distinct populations and displayed the phenotypic and functional characteristics of conventional B cells (88). Since the existence and function of NKB cells remain controversial, whether they are involved in IA pathogenesis is inclusive.

CD23^low^IgM^high^CD21^high^ MZB cells mainly reside on the border of the white pulp in the spleen and display reduced recirculatory potential. Autoantibodies against type II collagen (CII) play essential roles in murine arthritis development. Sandra Kleinau et al. showed that after CII immunization in DBA/1 mice, CII-autoreactive MZB cells expanded, were activated at the early stage and secreted large amounts of IgM that was reactive to autologous CII (89). Moreover, collagen-primed MZB cells displayed significant antigen-presenting capacities by inducing cognate T cell proliferation *in vitro* and IgG anti-collagen antibodies *in vivo*. The study highlighted autoreactive MZB cells as initiators that promote self-reactive responses in CIA (90).

B1 cells are the main producers of the T cell-independent antibodies IgM and IgA and are mainly located in the coelomic cavity (91). IL-10-producing CD1d^hi^CD5^+^ B cells, also defined as B10 cells, play an inhibitory role in arthritis development. Reduced B10 numbers were observed in PBMCs in RA and correlated with exacerbated disease activity (92, 93). Further *in vitro* studies of human B10 differentiation showed that STAT3 phosphorylation was indispensable for IL-10 production (94). B10 cells maintained immune tolerance by suppressing Th1/Th17 responses and inducing type 1 Treg cells in murine arthritis (95). Further mechanistic studies showed that B10 cells could directly inhibit Th17 cell generation by reducing STAT3 phosphorylation and RORγt expression (96). A recent study demonstrated a novel mechanism by which Bregs presented CD1d-lipid and induced iNKT cells to secrete IFN-γ, which in turn contributed to the down regulation of Th1 and Th17-adaptive immune responses and murine arthritis amelioration (97). In PBMCs and SF in PsA, B10 cells were decreased and inversely correlated with IL-17- and IFNγ-producing T cells (98). The percentage of CD24^hi^CD38^hi^ B10 cells was lower in the SF than in PBMCs of JIA patients (99). Collectively, these studies suggested a regulatory role of B10 cells through dampening adaptive Th1 and Th17 responses in IA.

## The Role of ILCs in IA

As described above, the three major groups in the ILC family mirror the canonical T helper subsets Th1, Th2, and Th17. The ILC family is involved in chronic inflammation. Conventional NK (cNK) cells are classified as ILC1s because they share the common transcription factor T-bet and produce a large amount of IFN-γ upon stimulation. ILC1s/NK cells are involved in the pathogenesis of chronic hepatitis B, tissue fibrosis and chronic viral diseases (100–102). ILC2s are associated with chronic human diseases, such as allergy and asthma, skin inflammation, and pulmonary fibrosis, which correlate with IL-33, IL-25, and type 2 cytokines (103–105). ILC3s are the dominant producers of IL-17 and IL-22 in the skin and intestine and are associated with chronic inflammation. The frequencies and cytokine production of ILC3s are increased in the skin of patients with psoriasis, Crohn’s disease and graft-versus-host disease (GVHD) (106–109). As IFN-γ, type 2 cytokines, IL-17A and IL-22 are all implicated in IA pathogenesis, a summary of the diversity and functions of ILCs in IA might help offer new strategies for targeting IA in the clinic.

Recently, a new member of the ILC family was introduced by Wang et al., regulatory ILCs (ILCregs), which have the CD45^+^Lin^–^CD127^+^IL-10^+^ phenotype (110). ILCregs reside in murine and human intestines and specifically produce IL-10 and transforming growth factor-β (TGF-β). Autocrine TGF-β is required for the expansion but not development of these cells. Although ILCregs produce similar cytokines to regulatory T cells (Tregs), they are defined by the distinct transcription factors *Id3* and *Sox4* but not *Foxp3*. As ILCregs are newly discovered and their roles in inflammation resolution in allergic airway inflammation or renal ischemia/reperfusion injury are limited (111, 112), whether these cells are involved in IA remains to be studied.

## NK Cells

Natural killer cells are essential components of the innate immune system and were originally characterized by their ability to kill tumor or virus-infected cells by directly releasing perforin- and granzyme-containing cytotoxic granules (113). Type I IFN, IL-2, IL-12, IL-15, and IL-18 are important cytokines for NK cell activation. Upon stimulation, NK cells can secrete cytokines such as IFN-γ, TNF-α, IL-5, IL-10, IL-13, and GM-CSF and the chemokines CCL3, CCL4, CCL5, and CXCL8 (114). In humans, NK cells can be classified into two major subtypes: CD56^bright^ and CD56^dim^ NK cells. CD56^dim^ NK cells exhibit relatively reduced cytotoxicity with increased production of cytokines that are predominately found in the peripheral blood. CD56^bright^ NK cells highly express CCR7 and CD62L to promote homing to secondary lymphoid organs. CD56^bright^ NK cells are tissue-resident and mainly reside in different tissues (e.g., lymph nodes, tonsils, liver, and uterus) (115).

Infiltrating tissue-resident NK cells have been detected in IA patients. Both seropositive RA and PsA patients showed decreased NK cell counts, while AS patients had higher percentages of NK cells in the peripheral blood than healthy controls (116–119). Infiltrating CD56^bright^ NK cells were observed in inflamed joints with high expression of activation markers (CD69 and NKp44) and enhanced TNF-α production regulated by CD94/NKG2A compared to those of PBMC subsets (120). IL-17-producing NK cells preferentially proliferate in the SF of active arthritis and undifferentiated SpA (121). An increased number of NKp44^+^ NK cells was detected in the inflamed gut of AS patients (122).

Natural killer cells were suggested to be potential promoters of bone destruction, T cell responses and FLS proliferation in RA. Kalle et al. discovered that NK cell depletion before murine CIA induction reduced the severity of arthritis and almost completely prevented the destruction of bone (123). Murine synovial NK cells express M-CSF and RANKL, which trigger monocyte differentiation into osteoclasts when NK cells are co-cultured with monocytes *in vitro* (123). SF from RA or PsA but not OA patients induced monocytes to differentiate into DCs in presence of NK cell-derived GM-CSF and CD154 (124). IL-22-secreting NK cell (NK-22) proportions were increased in SF compared with PBMCs from the same RA patient. Increased NK-22 cells can secrete IL-22 and TNF-α to promote RA FLS proliferation *in vitro* (125). However, in study performed by Jianmei W. et al. showed a regulatory role of NK cells in a murine CIA model. The researchers observed delayed arthritis progression with enhanced elimination of pathogenic Tfh and Th17 cells after activation of NK cells through blocking the inhibitory NKG2A/CD94 receptor (126). We reasoned that the contribution of NK cells to RA may differ from that of other NK subsets.

In PsA, NK cells contribute to disease amplification and persistence. Chiara et al. showed that NK cells infiltrated the psoriatic skin and exhibited the CD56^bright^CD16^–^ phenotype in PsA patients. The group further found that IL-2-primed NK cells released a large amount of IFN-γ, which can induce psoriatic keratinocyte activation and promote keratinocyte secretion of CXCL10 and CCL5 *in vitro* (127).

The role of NK cells in the pathogenesis of SpA remains controversial. A previous study demonstrated a tissue-protective role of NKp44^+^IL-22-producing cells in the gut tissue of AS patients (122). However, another study showed that circulating CD56^bright^ NK cells in AS patients promoted TNF-α secretion by autologous monocytes, which contributed to a worsened disease status (128). NK cells were thought to play a regulatory role in sJIA, and the dysfunction of these cells in sJIA was strongly associated with macrophage activation syndrome (MAS) (129). In a mouse model of sJIA, NK cell depletion or blockade of the NK cell activating receptor NKG2D increased the severity of sJIA-like symptoms, as well as increased the number of activated inflammatory monocytes, further indicating a regulatory role for NK cells in sJIA (130).

## ILC1s

Although they share the common feature of Tbet^+^IFNγ^+^ expression with NK cells, ILC1s express IL-7R and do not express cytotoxicity-related molecules, including perforin and granzyme B (131). A recent study showed that ILC1s played a critical role in viral infection through the rapid production of interferon (IFN)- γ, which occurs even earlier than in NK cells (132, 133). Moreover, unlike the capacity of NK cells to recirculate throughout the body, ILC1s appear to be tissue-resident. ILC1 populations were present in the SF and synovial tissue in inflamed RA, PsA and SpA patients (134). The frequency of ILC1s in the SF and synovial tissue was significantly increased compared with that of PBMCs from SpA patients (135). ILC1 frequency was significantly increased in RA or in individuals at risk of RA compared with that of controls, indicating a potential role of ILC1s in RA pathogenesis (136).

## ILC2s

Innate lymphoid cell2s express the type 2 T helper (T_*H*_2) cell-associated transcription factor GATA-binding factor 3 (GATA3) and cytokines, including IL-4, IL-5, and IL-13 (137). The role of ILC2s in IA might differ from different cytokine-secreting ILC2 subsets. ILC2 numbers were significantly higher in the peripheral blood of RA patients than in HCs but were inversely correlated with disease activity. Adoptive transfer of ILC2s attenuated murine arthritis severity, and ILC2-derived IL-4/13 inhibited IL-1β and TNFα secretion by macrophages, which indicated the immunoregulatory function of the IL-4/13-producing ILC2 subset in RA (138). However, in another study using the SKG model of autoimmune arthritis, Keiji Hirota et al. demonstrated that IL-2, IL33, or TLR9 ligands released from damaged tissue-resident cells in inflamed synovia could stimulate ILC2s to produce GM-CSF. Furthermore, the researchers found that ILC2-secreted GM-CSF was crucial in initiating autoimmune murine arthritis (139).

## ILC3s and LTI Cells

ILC3s provide an innate source of IL-17A and IL-22 and depend on RORγt and AHR for development. ILC3s consist of two major subsets: NKp46^+^ ILC3s and LTi-like CCR6^+^NKp46^–^ ILC3s. A previous study showed that ILC3 populations were present in the SF and inflamed joints of IA patients (134). A pathogenic role of ILC3s in IA has been suggested in previous studies. The proportions of CCR6^+^ ILC3s in arthritic mice were significantly higher than those in non-arthritic mice after CIA induction. Moreover, CCR6^+^ ILC3s in arthritic mice produced higher levels of IL-17A and IL-22 than those from control mice. In RA patients, the CCR6^+^ ILC percentage in SF was positively correlated with the number of tender and swollen joints (140). NKp44^+^ ILCs were hardly detected in PBMCs and SF in RA patients but were abundant in SF in PsA patients. Moreover, CCR6 and NKp44 were co-expressed on IL-17A-producing ILCs in SF, and the number of circulating NKp44^+^CCR6^+^ ILCs among PBMCs was negatively correlated with the disease activity of PsA patients (141). ILC3s characterized as Lyn-Tbet^+^RORc^–^NKp44^+^IL-23R^+^ that produced high levels of IL-17A and IL-22 were significantly expanded in the gut, SF and bone marrow of AS patients (142). Notably, epithelial cell-derived IL-7 but not IL-23-induced LTi cells to differentiate into ILC3s in AS patients (142). Proinflammatory CX3CR1^+^CD59^+^TL1A^+^IL-23^+^ mononuclear phagocytes (MNPs) were present in the synovial and bone marrow samples of AS patients and exhibited the ability to induce ILC3 expansion and activation (143). However, understanding of ILC3s in IA is still limited and requires further investigation.

## Therapeutic Implications

Research on innate lymphocytes in IA has advanced our understanding of their roles in IA. Drugs that not only inhibit adaptive Th17 cells but also IL-17A-producing innate lymphocytes might be a useful therapeutic strategy in IA. For example, a recent study detected the expression and activation of the PI3Kδ-Akt-mTOR pathway in inflamed SpA synovial tissue. The authors identified a promising target by selectively inhibiting PI3Kδ (with a compound named seletalisib). Seletalisib suppressed IL-17A and IL-17F production by innate-like MAIT cells, γδ T cells and adaptive Th17 cells, thereby inhibiting downstream inflammation and tissue remodeling responses (144). Moreover, IL-17-producing innate-like T cells responded to IL-23 and IL-1β activation; therefore, targeting IL-23 might be a promising strategy for IA treatment. Recent studies using the IL-23 blocking reagent ustekinumab showed good responses in clinical AS and PsA treatment (145, 146). Finally, blocking ILC2-derived GM-CSF through anti-IL-33 mAbs might also be a promising strategy for clinical IA treatment.

## Conclusion

**FIGURE 1:**
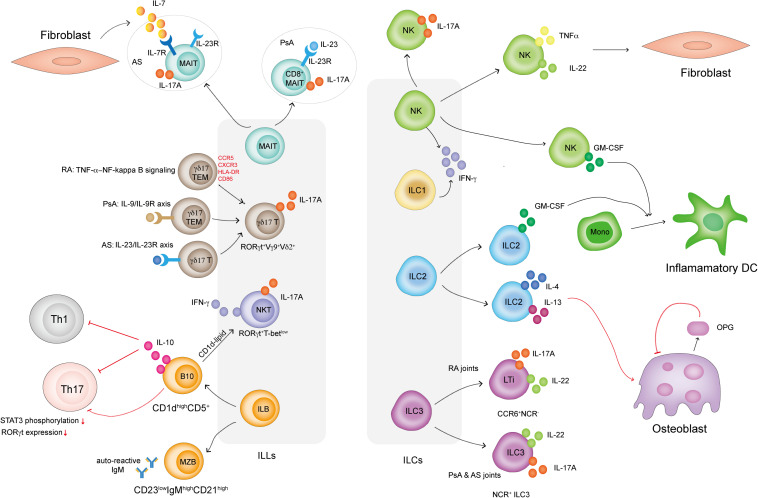
How ILLs and ILCs are involved in IA. MAIT cells in PsA patients display a CD8^+^IL-23R^+^ phenotype and respond to IL-23 to produce IL-17A. MAIT cells in AS patients display an IL-7R^+^IL-23R^+^ phenotype but only respond to FLS-derived IL-7 to produce IL-17A. γδ17 T cells in IA are RORγt^+^Vγ9^+^Vδ2^+^. γδ17 T cells in RA display the TEM phenotype and highly express CCR5, CXCR3, HLA-DR, and CD86. TNFα activates NF-κB signaling in γδ17 T cells and promotes IL-17A secretion. γδ17 T cells are activated via the IL-9/IL-9R and IL-23R/IL-23R axes in PsA and AS patients, respectively. NKT cells in IA are RORγt^+^T-bet^low^ and preferentially secrete IL-17A. ILB cells include CD23^low^CD21^high^ MZB cells, which secrete autoreactive IgM in IA, and CD1d^high^CD5^+^ B cells, which produce IL-10 to dampen Th1 and Th17 cell responses. B10 cells also directly inhibit Th17 cell responses by reducing STAT3 phosphorylation and RORγt expression. B10 cells present CD1d-lipids and induce iNKT cells to secrete IFN-γ. IL-17-producing NK cells are present in the SF in IA. NK cells also produce TNF-α and IL-22, which induce FLS proliferation. Both NK cells and ILC2s in IA secrete GM-CSF, which induces a proportion of monocytes to differentiate into inflammatory DCs. ILC2s in IA also produce IL-4 and IL-13, well-known Th2-related cytokines that induce osteoblasts to produce OPG. OPG is an inhibitor that prevents osteoclast formation. ILC3s can be classified into CCR6^+^NCR^–^ LTi cells and NCR^+^ ILC3 cells. Both subsets secrete IL-17A and IL-22. LTi cells mainly exist in RA joints, while NCR^+^ ILC3 cells can be found in the joints of PsA and AS patients.

A brief summary of ILLs and ILCs in IA is shown in Figure 1. The experimental evidence presented in this review indicated that both ILLs and ILCs might be important contributing sources of inflammatory cytokines in IA. Innate lymphocytes also affect adaptive immune responses and directly influence FLS and osteoclast proliferation, activation and function. Knowledge of the phenotype and detailed mechanisms of ILLs and ILCs in joints in IA is still limited. Deciphering the roles of ILLs and ILCs in IA initiation and development will ultimately provide insights into the mechanisms of IA and help design effective strategies for clinical treatments. Future work should focus on more in-depth studies about the functions of ILLs and ILCs in IA through genetically engineered mice and transcriptomic sequencing of patient samples.

## Author Contributions

XW conceived and wrote the manuscript.

## Conflict of Interest

The author declares that the research was conducted in the absence of any commercial or financial relationships that could be construed as a potential conflict of interest.

## References

[1] Ramiro S Radner H van der Heijde D Van Tubergen A Buchbinder R Aletaha D Combination therapy for pain management in inflammatory arthritis (rheumatoid arthritis, ankylosing spondylitis, psoriatic arthritis, other spondyloarthritis). *Cochr Database Syst Rev.* (2011) 5:CD008886. 10.1002/14651858.CD008886.pub2 PMC12416524 21975788

[2] Derksen V Huizinga T van der Woude D. The role of autoantibodies in the pathophysiology of rheumatoid arthritis. *Semin Immunopathol.* (2017) 39:437–46. 10.1007/s00281-017-0627-z 28451788 PMC5486798

[3] Ritchlin CT Colbert RA Gladman DD. Psoriatic arthritis. *N Engl J Med.* (2017) 376:957–70. 28273019 10.1056/NEJMra1505557

[4] Wen C Zheng Z Shao T Lin L Ehrlich SD Xie Z Chatelier EL Quantitative metagenomics reveals unique gut microbiome biomarkers in ankylosing spondylitis. *Genome Biol.* (2017) 18:142. 10.1186/s13059-017-1271-6 PMC5530561 28750650

[5] Dougados M Baeten D. Spondyloarthritis. *Lancet.* (2011) 377:2127–37. 21684383 10.1016/S0140-6736(11)60071-8

[6] Van Kaer L Postoak JL Wang C Yang G Wu LJC. Innate, innate-like and adaptive lymphocytes in the pathogenesis of MS and EAE. *Cell Mol Immunol.* (2019) 16:531–9. 10.1038/s41423-019-0221-5 30874627 PMC6804597

[7] Sun H Sun C Xiao W Sun R. Tissue-resident lymphocytes: from adaptive to innate immunity. *Cell Mol Immunol.* (2019) 16:205–15. 10.1038/s41423-018-0192-y 30635650 PMC6460493

[8] Sonnenberg GF Artis D. Innate lymphoid cells in the initiation, regulation and resolution of inflammation. *Nat Med.* (2015) 21:698. 10.1038/nm.3892 26121198 PMC4869856

[9] Buckley CD Gilroy DW Serhan CN Stockinger B Tak PP. The resolution of inflammation. *Nat Rev Immunol.* (2013) 13:59–66. 23197111 10.1038/nri3362

[10] Seillet C Jacquelot N. Sensing of physiological regulators by innate lymphoid cells. *Cell Mol Immunol.* (2019) 16:442–51. 10.1038/s41423-019-0217-1 30842626 PMC6474201

[11] Guo L Junttila IS Paul WE. Cytokine-induced cytokine production by conventional and innate lymphoid cells. *Trends Immunol.* (2012) 33:598–606. 10.1016/j.it.2012.07.006 22959641 PMC4799496

[12] Saferding V Blüml S. Innate immunity as the trigger of systemic autoimmune diseases. *J Autoimmun.* (2020) 110:102382. 10.1016/j.jaut.2019.102382 31883831

[13] Taylor PC Feldmann M. Anti-TNF biologic agents: still the therapy of choice for rheumatoid arthritis. *Nat Rev Rheumatol.* (2009) 5:578. 10.1038/nrrheum.2009.181 19798034

[14] Lee A Qiao Y Grigoriev G Chen J Park-Min KH Park SH Tumor necrosis factor α induces sustained signaling and a prolonged and unremitting inflammatory response in rheumatoid arthritis synovial fibroblasts. *Arthritis Rheum.* (2013) 65:928–38. 10.1002/art.37853 23335080 PMC3618592

[15] Osta B Benedetti G Miossec P. Classical and paradoxical effects of TNF-α on bone homeostasis. *Front Immunol.* (2014) 5:48. 10.3389/fimmu.2014.00048 24592264 PMC3923157

[16] Barrat FJ Crow MK Ivashkiv LB. Interferon target-gene expression and epigenomic signatures in health and disease. *Nat Immunol.* (2019) 20:1574–83. 10.1038/s41590-019-0466-2 31745335 PMC7024546

[17] Cavalcanti YVN Brelaz MCA Neves JK Ferraz JC Pereira VR. Role of TNF-alpha, IFN-gamma, and IL-10 in the development of pulmonary tuberculosis. *Pulm Med.* (2012) 2012:745483. 10.1155/2012/745483 PMC3515941 23251798

[18] Lee SH Kwon JY Kim SY Jung K Cho ML. Interferon-gamma regulates inflammatory cell death by targeting necroptosis in experimental autoimmune arthritis. *Sci Rep.* (2017) 7:1–9. 28860618 10.1038/s41598-017-09767-0 PMC5579272

[19] Page CE Smale S Carty SM Amos N Lauder SN Goodfellow RM Interferon-γ inhibits interleukin-1β-induced matrix metalloproteinase production by synovial fibroblasts and protects articular cartilage in early arthritis. *Arthritis Res Ther.* (2010) 12:R49. 10.1186/ar2960 PMC2888198 20307272

[20] Kelchtermans H Schurgers E Geboes L Mitera T Van Damme J Van Snick J Effector mechanisms of interleukin-17 in collagen-induced arthritis in the absence of interferon-γ and counteraction by interferon-γ. *Arthritis Res Ther.* (2009) 11:R122. 10.1186/ar2787 PMC2745806 19686583

[21] Dai H Adamopoulos IE. Psoriatic arthritis under the influence of IFNγ. *Clin Immunol.* (2020) 218:108513. 10.1016/j.clim.2020.108513 32574710 PMC7595649

[22] Karonitsch T Beckmann D Dalwigk K Niederreiter B Studenic P Byrne RA Targeted inhibition of Janus kinases abates interfon gamma-induced invasive behaviour of fibroblast-like synoviocytes. *Rheumatology.* (2018) 57:572–7. 10.1093/rheumatology/kex426 29228301

[23] Cho W Kim Y Jeoung D-I Kim Y-M Choe J. IL-4 and IL-13 suppress prostaglandins production in human follicular dendritic cells by repressing COX-2 and mPGES-1 expression through JAK1 and STAT6. *Mol Immunol.* (2011) 48:966–72. 10.1016/j.molimm.2011.01.007 21277633

[24] Chen Z Andreev D Oeser K Krljanac B Hueber A Kleyer A Th2 and eosinophil responses suppress inflammatory arthritis. *Nat Commun.* (2016) 7:1–12. 10.1007/978-1-4939-2911-5_1 PMC4899615 27273006

[25] Yamada A Takami M Kawawa T Yasuhara R Zhao B Mochizuki A Interleukin−4 inhibition of osteoclast differentiation is stronger than that of interleukin−13 and they are equivalent for induction of osteoprotegerin production from osteoblasts. *Immunology.* (2007) 120:573–9. 10.1111/j.1365-2567.2006.02538.x 17343616 PMC2265899

[26] Fujii T Kitaura H Kimura K Hakami ZW Takano-Yamamoto T. IL-4 inhibits TNF-α-mediated osteoclast formation by inhibition of RANKL expression in TNF-α-activated stromal cells and direct inhibition of TNF-α-activated osteoclast precursors via a T-cell-independent mechanism in vivo. *Bone.* (2012) 51:771–80. 10.1016/j.bone.2012.06.024 22776139

[27] Kundu-Raychaudhuri S Abria C Raychaudhuri SP. IL-9, a local growth factor for synovial T cells in inflammatory arthritis. *Cytokine.* (2016) 79:45–51. 10.1016/j.cyto.2015.12.020 26751012

[28] Chowdhury K Kumar U Das S Chaudhuri J Kumar P Kanjilal M Synovial IL-9 facilitates neutrophil survival, function and differentiation of Th17 cells in rheumatoid arthritis. *Arthritis Res Ther.* (2018) 20:18. 10.1186/s13075-017-1505-8 PMC5791733 29382374

[29] Guggino G Ciccia F Di Liberto D Lo Pizzo M Ruscitti P Cipriani P Interleukin (IL)−9/IL−9R axis drives γδ T cells activation in psoriatic arthritis patients. *Clin Exp Immunol.* (2016) 186:277–83. 10.1111/cei.12853 27543964 PMC5108067

[30] Rauber S Luber M Weber S Maul L Soare A Wohlfahrt T Resolution of inflammation by interleukin-9-producing type 2 innate lymphoid cells. *Nat Med.* (2017) 23:938. 10.1038/nm.4373 PMC5575995 28714991

[31] Fu W Hu W Shi L Mundra JJ Xiao G Dustin ML Foxo4-and Stat3-dependent IL-10 production by progranulin in regulatory T cells restrains inflammatory arthritis. *FASEB J.* (2017) 31:1354–67. 10.1096/fj.201601134r 28011648 PMC5349791

[32] Finnegan A Kaplan CD Cao Y Eibel H Glant TT Zhang J. Collagen-induced arthritis is exacerbated in IL-10-deficient mice. *Arthritis Res Ther.* (2002) 5:R18. 10.1186/ar601 PMC154422 12716449

[33] Ye L Wen Z Li Y Chen B Yu T Liu L Interleukin-10 attenuation of collagen-induced arthritis is associated with suppression of interleukin-17 and retinoid-related orphan receptor γt production in macrophages and repression of classically activated macrophages. *Arthritis Res Ther.* (2014) 16:R96. 10.1186/ar4544 PMC4060547 24742125

[34] Heo Y-J Joo Y-B Oh H-J Park M-K Heo Y-M Cho M-L IL-10 suppresses Th17 cells and promotes regulatory T cells in the CD4+ T cell population of rheumatoid arthritis patients. *Immunol Lett.* (2010) 127:150–6. 10.1016/j.imlet.2009.10.006 19895848

[35] Lubberts E. The IL-23–IL-17 axis in inflammatory arthritis. *Nat Rev Rheumatol.* (2015) 11:415. 10.1038/nrrheum.2015.53 25907700

[36] Pickens SR Volin MV Mandelin AM Kolls JK Pope RM Shahrara S. IL-17 contributes to angiogenesis in rheumatoid arthritis. *J Immunol.* (2010) 184:3233–41. 10.4049/jimmunol.0903271 20173024 PMC2857761

[37] Shahrara S Pickens SR Dorfleutner A Pope RM. IL-17 induces monocyte migration in rheumatoid arthritis. *J Immunol.* (2009) 182:3884–91. 10.4049/jimmunol.0802246 19265168 PMC2811490

[38] Koenders MI Kolls JK Oppers-Walgreen B Van Den Bersselaar L Joosten LA Schurr JR Interleukin−17 receptor deficiency results in impaired synovial expression of interleukin−1 and matrix metalloproteinases 3, 9, and 13 and prevents cartilage destruction during chronic reactivated streptococcal cell wall–induced arthritis. *Arthritis Rheum.* (2005) 52:3239–47. 10.1002/art.21342 16200598

[39] Ganesan R Rasool M. Interleukin 17 regulates SHP-2 and IL-17RA/STAT-3 dependent Cyr61, IL-23 and GM-CSF expression and RANKL mediated osteoclastogenesis by fibroblast-like synoviocytes in rheumatoid arthritis. *Mol Immunol.* (2017) 91:134–44. 10.1016/j.molimm.2017.09.003 28898718

[40] Raychaudhuri SP Raychaudhuri SK Genovese MC. IL-17 receptor and its functional significance in psoriatic arthritis. *Mol Cell Biochem.* (2012) 359:419–29. 10.1007/s11010-011-1036-6 21894442

[41] Samarpita S Doss HM Ganesan R Rasool M. Interleukin 17 under hypoxia mimetic condition augments osteoclast mediated bone erosion and expression of HIF-1α and MMP-9. *Cell Immunol.* (2018) 332:39–50. 10.1016/j.cellimm.2018.07.005 30029761

[42] Lemos HP Grespan R Vieira SM Cunha TM Verri WA Fernandes KS Prostaglandin mediates IL-23/IL-17-induced neutrophil migration in inflammation by inhibiting IL-12 and IFNγ production. *Proc Natl Acad Sci USA.* (2009) 106:5954–9. 10.1073/pnas.0812782106 19289819 PMC2667068

[43] Carrión M Juarranz Y Martínez C González-Álvaro I Pablos JL Gutiérrez-Cañas I IL-22/IL-22R1 axis and S100A8/A9 alarmins in human osteoarthritic and rheumatoid arthritis synovial fibroblasts. *Rheumatology.* (2013) 52:2177–86. 10.1093/rheumatology/ket315 24056519

[44] Mitra A Raychaudhuri SK Raychaudhuri SP. Functional role of IL-22 in psoriatic arthritis. *Arthritis Res Ther.* (2012) 14:R65. 10.1186/ar3781 PMC3446433 22417743

[45] Kim KW Kim HR Park JY Park JS Oh HJ Woo YJ Interleukin−22 promotes osteoclastogenesis in rheumatoid arthritis through induction of RANKL in human synovial fibroblasts. *Arthritis Rheum.* (2012) 64:1015–23. 10.1002/art.33446 22034096

[46] Pinto LG Talbot J Peres RS Franca RF Ferreira SH Ryffel B Joint production of IL-22 participates in the initial phase of antigen-induced arthritis through IL-1β production. *Arthritis Res Ther.* (2015) 17:235. 10.1186/s13075-015-0759-2 PMC4556214 26330334

[47] Corneth OB Reijmers RM Mus AM Asmawidjaja PS van Hamburg JP Papazian N Loss of IL−22 inhibits autoantibody formation in collagen−induced arthritis in mice. *Eur J Immunol.* (2016) 46:1404–14. 10.1002/eji.201546241 27067635

[48] Zhao W Zhao G Wang B. Revisiting GM-CSF as an adjuvant for therapeutic vaccines. *Cell Mol Immunol.* (2018) 15:187–9. 10.1038/cmi.2017.105 29057973 PMC5811680

[49] Cook AD Pobjoy J Steidl S Dürr M Braine EL Turner AL Granulocyte-macrophage colony-stimulating factor is a key mediator in experimental osteoarthritis pain and disease development. *Arthritis Res Ther.* (2012) 14:R199. 10.1186/ar4037 PMC3580511 22995428

[50] Guo X Higgs BW Bay-Jensen A-C Wu Y Karsdal MA Kuziora M Blockade of GM-CSF pathway induced sustained suppression of myeloid and T cell activities in rheumatoid arthritis. *Rheumatology.* (2018) 57:175–84. 10.1093/rheumatology/kex383 29069507

[51] Reynolds G Gibbon J Pratt A Wood M Coady D Raftery G Synovial CD4+ T-cell-derived GM-CSF supports the differentiation of an inflammatory dendritic cell population in rheumatoid arthritis. *Ann Rheum Dis.* (2016) 75:899–907. 10.1136/annrheumdis-2014-206578 25923217 PMC4853576

[52] Jang J Lim D-S Choi Y-E Jeong Y Yoo S-A Kim W-U MLN51and GM-CSF involvement in the proliferation of fibroblast-like synoviocytes in the pathogenesis of rheumatoid arthritis. *Arthritis Res Ther.* (2006) 8:R170. 10.1186/ar2079 PMC1794514 17101062

[53] Croxford AL Lanzinger M Hartmann FJ Schreiner B Mair F Pelczar P The cytokine GM-CSF drives the inflammatory signature of CCR2+ monocytes and licenses autoimmunity. *Immunity.* (2015) 43:502–14. 10.1016/j.immuni.2015.08.010 26341401

[54] Fujii S-I Shimizu K. Immune networks and therapeutic targeting of iNKT cells in cancer. *Trends Immunol.* (2019) 40:984–97. 10.1016/j.it.2019.09.008 31676264

[55] Linsen L Thewissen M Baeten K Somers V Geusens P Raus J Peripheral blood but not synovial fluid natural killer T cells are biased towards a Th1-like phenotype in rheumatoid arthritis. *Arthritis Res Ther.* (2005) 7:R493. 10.1186/ar1695 PMC1174940 15899036

[56] Zhao M Svensson MN Venken K Chawla A Liang S Engel I Altered thymic differentiation and modulation of arthritis by invariant NKT cells expressing mutant ZAP70. *Nat Commun.* (2018) 9:1–17. 29980684 10.1038/s41467-018-05095-7 PMC6035278

[57] Chiba A Kaieda S Oki S Yamamura T Miyake S. The involvement of Vα14 natural killer T cells in the pathogenesis of arthritis in murine models. *Arthritis Rheum.* (2005) 52:1941–8. 10.1002/art.21056 15934073

[58] Kim HY Kim HJ Min HS Kim S Park WS Park SH NKT cells promote antibody-induced joint inflammation by suppressing transforming growth factor β1 production. *J Exp Med.* (2005) 201:41–7. 10.1084/jem.20041400 15630137 PMC2212773

[59] Jung S Shin HS Hong C Lee H Park Y-K Shin JH Natural killer T cells promote collagen-induced arthritis in DBA/1 mice. *Biochem Biophys Res Commun.* (2009) 390:399–403. 10.1016/j.bbrc.2009.09.008 19737532

[60] Teige A Bockermann R Hasan M Olofsson KE Liu Y Issazadeh-Navikas S. CD1d-dependent NKT cells play a protective role in acute and chronic arthritis models by ameliorating antigen-specific Th1 responses. *J Immunol.* (2010) 185:345–56. 10.4049/jimmunol.0901693 20525883

[61] Peternel S Kaštelan M. Immunopathogenesis of psoriasis: focus on natural killer T cells. *J Eur Acad Dermatol Venereol.* (2009) 23:1123–7. 10.1111/j.1468-3083.2009.03292.x 19453772

[62] Venken K Jacques P Mortier C Labadia ME Decruy T Coudenys J Rorγt inhibition selectively targets IL-17 producing iNKT and γδ-T cells enriched in spondyloarthritis patients. *Nat Commun.* (2019) 10:1–15. 30602780 10.1038/s41467-018-07911-6 PMC6315029

[63] Kim T Lee S Cho Y Park S Jin H Kim M Immune cells and bone formation in ankylosing spondylitis. *Clin Exp Rheumatol.* (2012) 30:469. 22510234

[64] Elewaut D. NKT and related cells: key roles in spondyloarthritis? *Ann Rheum Dis.* (2013) 72:A29–29.

[65] Godfrey DI Koay H-F McCluskey J Gherardin NA. The biology and functional importance of MAIT cells. *Nat Immunol.* (2019) 20:1110–28. 10.1038/s41590-019-0444-8 31406380

[66] Koppejan H Jansen DT Hameetman M Thomas R Toes RE van Gaalen FA Altered composition and phenotype of mucosal-associated invariant T cells in early untreated rheumatoid arthritis. *Arthritis Res Ther.* (2019) 21:3. 10.1186/s13075-018-1799-1 PMC6321723 30611306

[67] Menon B Gullick NJ Walter GJ Rajasekhar M Garrood T Evans HG Interleukin−17+ CD8+ T cells are enriched in the joints of patients with psoriatic arthritis and correlate with disease activity and joint damage progression. *J Arthritis Rheumatol.* (2014) 66:1272–81. 10.1002/art.38376 24470327 PMC4158887

[68] Gracey E Qaiyum Z Almaghlouth I Lawson D Karki S Avvaru N IL-7 primes IL-17 in mucosal-associated invariant T (MAIT) cells, which contribute to the Th17-axis in ankylosing spondylitis. *Ann Rheum Dis.* (2016) 75:2124–32. 10.1136/annrheumdis-2015-208902 27165176

[69] Kim M Yoo S-J Kang SW Kwon J Choi I Lee CH. TNF α and IL-1β in the synovial fluid facilitate mucosal-associated invariant T (MAIT) cell migration. *Cytokine.* (2017) 99:91–8. 10.1016/j.cyto.2017.07.007 28756336

[70] Chiba A Tajima R Tomi C Miyazaki Y Yamamura T Miyake S. Mucosal−associated invariant T cells promote inflammation and exacerbate disease in murine models of arthritis. *Arthritis Rheum.* (2012) 64:153–61. 10.1002/art.33314 21904999

[71] Raychaudhuri SK Abria C Mitra A Raychaudhuri SP. Functional significance of MAIT cells in psoriatic arthritis. *Cytokine.* (2020) 125:154855. 10.1016/j.cyto.2019.154855 31541902

[72] Nguyen CT Maverakis E Eberl M Adamopoulos IE. γδ T cells in rheumatic diseases: from fundamental mechanisms to autoimmunity. *Semin Immunopathol.* (2019) 41:595–605. 10.1007/s00281-019-00752-5 31506867 PMC6815259

[73] Cai Y Fleming C Yan J. New insights of T cells in the pathogenesis of psoriasis. *Cell Mol Immunol.* (2012) 9:302–9. 10.1038/cmi.2012.15 22705915 PMC4132586

[74] McKenzie DR Kara EE Bastow CR Tyllis TS Fenix KA Gregor CE IL-17-producing γδ T cells switch migratory patterns between resting and activated states. *Nat Commun.* (2017) 8:1–13. 28580944 10.1038/ncomms15632 PMC5465362

[75] Sutton CE Lalor SJ Sweeney CM Brereton CF Lavelle EC Mills KH. Interleukin-1 and IL-23 induce innate IL-17 production from γδ T cells, amplifying Th17 responses and autoimmunity. *Immunity.* (2009) 31:331–41. 10.1016/j.immuni.2009.08.001 19682929

[76] Ramírez-Valle F Gray EE Cyster JG. Inflammation induces dermal Vγ4+ γδT17 memory-like cells that travel to distant skin and accelerate secondary IL-17–driven responses. *Proc Natl Acad Sci USA.* (2015) 112:8046–51. 10.1073/pnas.1508990112 26080440 PMC4491769

[77] Sutton CE Mielke LA Mills KH. IL-17-producing γδ T cells and innate lymphoid cells. *Eur J Immunol.* (2012) 42:2221–31. 10.1002/eji.201242569 22949320

[78] Roark CL French JD Taylor MA Bendele AM Born WK O’Brien RL. Exacerbation of collagen-induced arthritis by oligoclonal, IL-17-producing γδ T cells. *J Immunol.* (2007) 179:5576–83. 10.4049/jimmunol.179.8.5576 17911645 PMC2768546

[79] Akitsu A Ishigame H Kakuta S Chung SH Ikeda S Shimizu K. IL-1 receptor antagonist-deficient mice develop autoimmune arthritis due to intrinsic activation of IL-17-producing CCR2+ Vγ6+ γδ T cells. *Nat Commun.* (2015) 6:7464. 10.1038/ncomms8464 PMC4521288 26108163

[80] Ito Y Usui T Kobayashi S Iguchi-Hashimoto M Ito H Yoshitomi H Gamma/delta T cells are the predominant source of interleukin-17 in affected joints in collagen-induced arthritis, but not in rheumatoid arthritis. *Arthritis Rheum.* (2009) 60:2294–303. 10.1002/art.24687 19644886

[81] Mo W-X Yin S-S Chen H Zhou C Zhou J-X Zhao L-D Chemotaxis of Vδ2 T cells to the joints contributes to the pathogenesis of rheumatoid arthritis. *Ann Rheum Dis.* (2017) 76:2075–84. 10.1136/annrheumdis-2016-211069 28866647 PMC5705844

[82] Hu C Qian L Miao Y Huang Q Miao P Wang P Antigen-presenting effects of effector memory Vγ9Vδ2 T cells in rheumatoid arthritis. *Cell Mol Immunol.* (2012) 9:245–54. 10.1038/cmi.2011.50 22139198 PMC4012843

[83] Kenna TJ Davidson SI Duan R Bradbury LA McFarlane J Smith M Enrichment of circulating interleukin−17–secreting interleukin−23 receptor–positive γ/δ T cells in patients with active ankylosing spondylitis. *Arthritis Rheum.* (2012) 64:1420–9. 10.1002/art.33507 22144400

[84] Gaur P Misra R Aggarwal A. Natural killer cell and gamma delta T cell alterations in enthesitis related arthritis category of juvenile idiopathic arthritis. *Clin Immunol.* (2015) 161:163–9. 26244610 10.1016/j.clim.2015.07.012

[85] Jackson-Jones LH Bénézech C. Control of innate-like B cell location for compartmentalised IgM production. *Curr Opin Immunol.* (2018) 50:9–13. 29078198 10.1016/j.coi.2017.10.006

[86] Geherin SA Gómez D Glabman RA Ruthel G Hamann A Debes GF. IL-10+ innate-like B cells are part of the skin immune system and require α4β1 integrin to migrate between the peritoneum and inflamed skin. *J Immunol.* (2016) 196:2514–25. 26851219 10.4049/jimmunol.1403246 PMC4779667

[87] Wang S Xia P Chen Y Huang G Xiong Z Liu J Natural killer-like B cells prime innate lymphocytes against microbial infection. *Immunity.* (2016) 45:131–44. 27421702 10.1016/j.immuni.2016.06.019

[88] Kerdiles YM Almeida FF Thompson T Chopin M Vienne M Bruhns P Natural-killer-like B cells display the phenotypic and functional characteristics of conventional B cells. *Immunity.* (2017) 47:199–200. 28813647 10.1016/j.immuni.2017.07.026 PMC5705200

[89] Carnrot C Prokopec KE Råsbo K Karlsson MC Kleinau S. Marginal zone B cells are naturally reactive to collagen type II and are involved in the initiation of the immune response in collagen-induced arthritis. *Cell Mol Immunol.* (2011) 8:296–304. 21358667 10.1038/cmi.2011.2 PMC4002445

[90] Palm A-KE Friedrich HC Mezger A Salomonsson M Myers LK Kleinau S. Function and regulation of self-reactive marginal zone B cells in autoimmune arthritis. *Cell Mol Immunol.* (2015) 12:493–504. 25958842 10.1038/cmi.2015.37 PMC4496548

[91] Wang X Ye C Lin X Ma K Xiao F Dong L New insights into the significance of the BCR repertoire in B-1 cell development and function. *Cell Mol Immunol.* (2019) 16:772–3. 10.1038/s41423-019-0249-6 31197257 PMC6804932

[92] Xu L Liu X Liu H Zhu L Zhu H Zhang J Impairment of granzyme B-producing regulatory B cells correlates with exacerbated rheumatoid arthritis. *Front Immunol.* (2017) 8:768. 10.3389/fimmu.2017.00768 28713386 PMC5491972

[93] Ma L Liu B Jiang Z Jiang Y. Reduced numbers of regulatory B cells are negatively correlated with disease activity in patients with new-onset rheumatoid arthritis. *Clin Rheumatol.* (2014) 33:187–95. 10.1007/s10067-013-2359-3 23949637

[94] Bankó Z Pozsgay J Szili D Tóth M Gáti T Nagy G Induction and differentiation of IL-10–producing regulatory B cells from healthy blood donors and rheumatoid arthritis patients. *J Immunol.* (2017) 198:1512–20. 10.4049/jimmunol.1600218 28087671

[95] Carter NA Rosser EC Mauri C. Interleukin-10 produced by B cells is crucial for the suppression of Th17/Th1 responses, induction of T regulatory type 1 cells and reduction of collagen-induced arthritis. *Arthritis Res Ther.* (2012) 14:R32. 10.1186/ar3736 22315945 PMC3392827

[96] Yang M Deng J Liu Y Ko K-H Wang X Jiao Z IL-10–producing regulatory B10 cells ameliorate collagen-induced arthritis via suppressing Th17 cell generation. *Am J Pathol.* (2012) 180:2375–85. 10.1016/j.ajpath.2012.03.010 22538089

[97] Oleinika K Rosser E Matei D Nistala K Bosma A Drozdov I CD1d-dependent immune suppression mediated by regulatory B cells through modulations of iNKT cells. *Nat Commun.* (2018) 9:1–17. 10.1038/s41467-018-02911-y 29449556 PMC5814456

[98] Mavropoulos A Varna A Zafiriou E Liaskos C Alexiou I Roussaki-Schulze A IL-10 producing Bregs are impaired in psoriatic arthritis and psoriasis and inversely correlate with IL-17-and IFNγ-producing T cells. *Clin Immunol.* (2017) 184:33–41. 10.1016/j.clim.2017.04.010 28461105

[99] Zhao Q Jung LK. Frequency of CD19+ CD24 hi CD38 hi regulatory B cells is decreased in peripheral blood and synovial fluid of patients with juvenile idiopathic arthritis: a preliminary study. *Pediatr Rheumatol.* (2018) 16:44. 10.1186/s12969-018-0262-9 29973221 PMC6033228

[100] Yang Z Tang T Wei X Yang S Tian Z. Type 1 innate lymphoid cells contribute to the pathogenesis of chronic hepatitis B. *Innate Immun.* (2015) 21:665–73. 10.1177/1753425915586074 25977358

[101] Wang H Shen L Sun X Liu F Feng W Jiang C Adipose group 1 innate lymphoid cells promote adipose tissue fibrosis and diabetes in obesity. *Nat Commun.* (2019) 10:1–14. 10.1038/s41467-019-11270-1 31332184 PMC6646407

[102] Peng H Tian Z. NK cells in liver homeostasis and viral hepatitis. *Sci China Life Sci.* (2018) 61:1477–85. 10.1007/s11427-018-9407-2 30421296

[103] Gour N Smole U Yong H-M Lewkowich IP Yao N Singh A C3a is required for ILC2 function in allergic airway inflammation. *Mucosal Immunol.* (2018) 11:1653–62. 10.1038/s41385-018-0064-x 30104625 PMC6279480

[104] Moretti S Renga G Oikonomou V Galosi C Pariano M Iannitti RG A mast cell-ILC2-Th9 pathway promotes lung inflammation in cystic fibrosis. *Nat Commun.* (2017) 8:1–13. 10.1038/ncomms14017 28090087 PMC5241810

[105] Mjösberg J Eidsmo L. Update on innate lymphoid cells in atopic and non−atopic inflammation in the airways and skin. *Clin Exp Allergy.* (2014) 44:1033–43. 10.1111/cea.12353 24912880

[106] Villanova F Flutter B Tosi I Grys K Sreeneebus H Perera GK Characterization of innate lymphoid cells in human skin and blood demonstrates increase of NKp44+ ILC3 in psoriasis. *J Invest Dermatol.* (2014) 134:984–91. 10.1038/jid.2013.477 24352038 PMC3961476

[107] Teunissen MB Munneke JM Bernink JH Spuls PI Te Velde A Cheuk S Composition of innate lymphoid cell subsets in the human skin: enrichment of NCR+ ILC3 in lesional skin and blood of psoriasis patients. *J Invest Dermatol.* (2014) 134:2351–60. 10.1038/jid.2014.146 24658504

[108] Lo BC Gold MJ Hughes MR Antignano F Valdez Y Zaph C The orphan nuclear receptor ROR alpha and group 3 innate lymphoid cells drive fibrosis in a mouse model of Crohn’s disease. *Sci Immunol.* (2016) 1:eaaf8864 10.1126/sciimmunol.aaf8864 PMC5489332 28670633

[109] Hazenberg MD Haverkate NJ van Lier YF Spits H Krabbendam L Bemelman WA Human ectoenzyme-expressing ILC3: immunosuppressive innate cells that are depleted in graft-versus-host disease. *Blood Adv.* (2019) 3:3650–60. 10.1182/bloodadvances.2019000176 31751473 PMC6880892

[110] Wang S Xia P Chen Y Qu Y Xiong Z Ye B Regulatory innate lymphoid cells control innate intestinal inflammation. *Cell.* (2017) 171:201–16.e218. 10.1016/j.cell.2017.07.027 28844693

[111] Morita H Kubo T Rückert B Ravindran A Soyka MB Rinaldi AO Induction of human regulatory innate lymphoid cells from group 2 innate lymphoid cells by retinoic acid. *J Allergy Clin Immunol.* (2019) 143:2190–201.e2199. 10.1016/j.jaci.2018.12.1018 30682454

[112] Cao Q Wang R Wang Y Niu Z Chen T Wang C Regulatory innate lymphoid cells suppress innate immunity and reduce renal ischemia/reperfusion injury. *Kidney Int.* (2020) 97:130–42. 10.1016/j.kint.2019.07.019 31685310

[113] Sivori S Vacca P Del Zotto G Munari E Mingari MC Moretta L. Human NK cells: surface receptors, inhibitory checkpoints, and translational applications. *Cell Mol Immunol.* (2019) 16:430–41. 10.1038/s41423-019-0206-4 30778167 PMC6474200

[114] Habif G Crinier A André P Vivier E Narni-Mancinelli E. Targeting natural killer cells in solid tumors. *Cell Mol Immunol.* (2019) 16:415–22. 10.1038/s41423-019-0224-2 30911118 PMC6474204

[115] Björkström NK Ljunggren H-G Michaelsson J. Emerging insights into natural killer cells in human peripheral tissues. *Nat Rev Immunol.* (2016) 16:310–20. 10.1038/nri.2016.34 27121652

[116] Conigliaro P Triggianese P Perricone C Chimenti M Di Muzio G Ballanti E Restoration of peripheral blood natural killer and B cell levels in patients affected by rheumatoid and psoriatic arthritis during etanercept treatment. *Clin Exp Immunol.* (2014) 177:234–43. 10.1111/cei.12335 24666401 PMC4089172

[117] Cameron A Kirby B Griffiths C. Circulating natural killer cells in psoriasis. *Br J Dermatol.* (2003) 149:160–4. 10.1046/j.1365-2133.2003.05319.x 12890211

[118] Azuz-Lieberman N Markel G Mizrahi SA Gazit R Hanna J Achdout H The involvement of NK cells in ankylosing spondylitis. *Int Immunol.* (2005) 17:837–45. 10.1093/intimm/dxh270 15937057

[119] Chalan P Bijzet J Kroesen B-J Boots AM Brouwer E. Altered natural killer cell subsets in seropositive arthralgia and early rheumatoid arthritis are associated with autoantibody status. *J Rheumatol.* (2016) 43:1008–16. 10.3899/jrheum.150644 27036380

[120] De Matos CT Berg L Michaëlsson J Felländer-Tsai L Kärre K Söderström K. Activating and inhibitory receptors on synovial fluid natural killer cells of arthritis patients: role of CD94/NKG2A in control of cytokine secretion. *J Immunol.* (2007) 122:291–301. 10.1111/j.1365-2567.2007.02638.x 17521371 PMC2266001

[121] Chowdhury AC Chaurasia S Mishra SK Aggarwal A Misra R. IL-17 and IFN-γ producing NK and γδ-T cells are preferentially expanded in synovial fluid of patients with reactive arthritis and undifferentiated spondyloarthritis. *J Clin Immunol.* (2017) 183:207–12. 10.1016/j.clim.2017.03.016 28390966

[122] Ciccia F Accardo-Palumbo A Alessandro R Rizzo A Principe S Peralta S Interleukin-22 and interleukin-22–producing NKp44+ natural killer cells in subclinical gut inflammation in ankylosing spondylitis. *Arthritis Rheum.* (2012) 64:1869–78. 10.1002/art.34355 22213179

[123] Söderström K Stein E Colmenero P Purath U Müller-Ladner U de Matos CT Natural killer cells trigger osteoclastogenesis and bone destruction in arthritis. *Proc Natl Acad Sci USA.* (2010) 107:13028–33. 10.1073/pnas.1000546107 20615964 PMC2919936

[124] Zhang AL Colmenero P Purath U Teixeira de Matos C Hueber W Klareskog L Natural killer cells trigger differentiation of monocytes into dendritic cells. *J Am Soc Hematol.* (2007) 110:2484–93. 10.1182/blood-2007-02-076364 17626840 PMC1988958

[125] Ren J Feng Z Lv Z Chen X Li J. Natural killer-22 cells in the synovial fluid of patients with rheumatoid arthritis are an innate source of interleukin 22 and tumor necrosis factor-α. *J Rheumatol.* (2011) 38:2112–8. 10.3899/jrheum.101377 21765110

[126] Leavenworth JW Wang X Wenander CS Spee P Cantor H. Mobilization of natural killer cells inhibits development of collagen-induced arthritis. *Proc Natl Acad Sci USA.* (2011) 108:14584–9. 10.1073/pnas.1112188108 21873193 PMC3167502

[127] Ottaviani C Nasorri F Bedini C de Pità O Girolomoni G Cavani A. CD56brightCD16–NK cells accumulate in psoriatic skin in response to CXCL10 and CCL5 and exacerbate skin inflammation. *Eur J Immunol.* (2006) 36:118–28. 10.1002/eji.200535243 16323244

[128] Yang M Zhou Y Liu L Wang S Jiang J Shang Q Decreased A20 expression on circulating CD56bright NK cells contributes to a worse disease status in patients with ankylosing spondylitis. *Clin Exp Immunol.* (2019) 198:1–10. 10.1111/cei.13341 31206174 PMC6718289

[129] Vandenhaute J Wouters CH Matthys P. Natural killer cells in systemic autoinflammatory diseases: a focus on systemic juvenile idiopathic arthritis and macrophage activation syndrome. *Front Immunol.* (2019) 10:3089. 10.3389/fimmu.2019.03089 32010140 PMC6974473

[130] Vandenhaute J Avau A Filtjens J Malengier-Devlies B Imbrechts M Van den Berghe N Regulatory role for NK cells in a mouse model of systemic juvenile idiopathic arthritis. *J Immunol.* (2019) 203:3339–48. 10.4049/jimmunol.1900510 31676671

[131] Kansler ER Li MO. Innate lymphocytes–lineage, localization and timing of differentiation. *Cell Mol Immunol.* (2019) 16:627–33. 10.1038/s41423-019-0211-7 30804475 PMC6804950

[132] Weizman OE Adams NM Schuster I Krishna C Pritykin Y Lau C ILC1 confer early host protection at initial sites of viral infection. *Cell.* (2017) 2017:S0092867417311832. 10.1016/j.cell.2017.09.052 29056343 PMC5687850

[133] Wang X Peng H Tian Z. Innate lymphoid cell memory. *Cell Mol Immunol.* (2019) 16:423–9. 10.1038/s41423-019-0212-6 30796350 PMC6474199

[134] Al-MossawiMManou-StathopoulouSDe WitJKendrickBGundleRBownessP. Identification and phenotyping of innate lymphoid cells present in the diseased joints of patients with spondyloarthritis, rheumatoid arthritis and psoriatic arthritis. *Proceedings of the Clinical And Experimental Rheumatology; 2014: Clinical 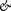 Exper Rheumatology Via Santa Maria 31, 56126*, PISA. (2014). p. 811–811.

[135] Yeremenko N Noordenbos T Blijdorp I Hreggvidsdottir H Germar K Bernink J *Human Type 1 Innate Lymphoid Cells Accumulate in the Inflamed Synovium in Spondyloarthritis.* London: BMJ Publishing Group Ltd (2015). 10.1136/annrheumdis-2015-eular.5907

[136] Rodríguez-Carrio J Hähnlein JS Ramwadhdoebe TH Semmelink J Choi I van Lienden K Altered Innate Lymphoid cells subsets in human lymph node biopsies during the at risk and earliest phase of rheumatoid arthritis. *Arthritis Rheumatol.* (2016) 69:70–6. 10.1002/art.39811 27428460 PMC6681066

[137] Gurram RK Zhu J. Orchestration between ILC2s and Th2 cells in shaping type 2 immune responses. *Cell Mol Immunol.* (2019) 16:225–35. 10.1038/s41423-019-0210-8 30792500 PMC6460501

[138] Omata Y Frech M Primbs T Lucas S Andreev D Scholtysek C Group 2 innate lymphoid cells attenuate inflammatory arthritis and protect from bone destruction in mice. *Cell Rep.* (2018) 24:169–80. 10.1016/j.celrep.2018.06.005 29972778

[139] Hirota K Hashimoto M Ito Y Matsuura M Ito H Tanaka M Autoimmune Th17 cells induced synovial stromal and innate lymphoid cell secretion of the cytokine GM-CSF to initiate and augment autoimmune arthritis. *Immunity.* (2018) 48:1220–32.e1225. 10.1016/j.immuni.2018.04.009 29802020 PMC6024031

[140] Takaki-Kuwahara A Arinobu Y Miyawaki K Yamada H Tsuzuki H Irino K CCR6+ group 3 innate lymphoid cells accumulate in inflamed joints in rheumatoid arthritis and produce Th17 cytokines. *Arthritis Res Ther.* (2019) 21:1–9. 10.1186/s13075-019-1984-x 31470891 PMC6716915

[141] Leijten E van Kempen T Boes M. Activated group 3 innate lymphoid cells are selectively enriched in psoriatic arthritis synovial fluid. *Arthritis Rheumatol.* (2015) 67:2673–8. 10.1002/art.39261 26137857

[142] Ciccia F Guggino G Rizzo A Saieva L Peralta S Giardina A Type 3 innate lymphoid cells producing IL-17 and IL-22 are expanded in the gut, in the peripheral blood, synovial fluid and bone marrow of patients with ankylosing spondylitis. *Ann Rheum Dis.* (2015) 74:1739–47. 10.1136/annrheumdis-2014-206323 25902790

[143] Ciccia F Guggino G Zeng M Thomas R Ranganathan V Rahman A Pro-inflammatory CX3CR1+ CD59+ TL1A+ IL-23+ monocytes are expanded in patients with Ankylosing Spondylitis and modulate ILC3 immune functions. *Arthritis Rheumatol.* (2018) 70:2003–13. 10.1002/art.40582 29869839

[144] Chen S Paveley R Kraal L Sritharan L Stevens E Dedi N Selective targeting of PI3Kδ suppresses human IL-17-producing T cells and innate-like lymphocytes and may be therapeutic for IL-17-mediated diseases. *J Autoimmun.* (2020) 2020:102435. 10.1016/j.jaut.2020.102435 32360069

[145] McInnes IB Kavanaugh A Gottlieb AB Puig L Rahman P Ritchlin C Efficacy and safety of ustekinumab in patients with active psoriatic arthritis: 1 year results of the phase 3, multicentre, double-blind, placebo-controlled PSUMMIT 1 trial. *Lancet.* (2013) 382:780–9. 10.1016/S0140-6736(13)60594-2 23769296

[146] Poddubnyy D Hermann K-GA Callhoff J Listing J Sieper J. Ustekinumab for the treatment of patients with active ankylosing spondylitis: results of a 28-week, prospective, open-label, proof-of-concept study (TOPAS). *Ann Rheum Dis.* (2014) 73:817–23. 10.1136/annrheumdis-2013-204248 24389297

